# Knowledge and participation in exercise and physical activity among pregnant women in Ho, Ghana

**DOI:** 10.3389/fpubh.2022.927191

**Published:** 2022-11-16

**Authors:** Drusilla Obenewaa Asante, Francis Osei, Fridaus Abdul-Samed, Victoria Dzifa Nanevie

**Affiliations:** ^1^Department of Sports and Exercise Medical Sciences, School of Sports and Exercise Medicine, University of Health and Allied Science, Ho, Ghana; ^2^Medical Sociology and Psychobiology, Department of Health and Physical Activity, University of Potsdam, Potsdam, Germany

**Keywords:** physical activity, barriers to physical activity, education, pregnancy, gestational age

## Abstract

**Background:**

Physical activity (PA) and exercise have been identified to improve the general fitness and health. Although, the Pregnancy Physical Activity Questionnaire (PPAQ) has been validated for use in assessing PA in pregnant women. However, understanding the knowledge and participation levels of PA in pregnant women in the underdeveloped regions of Ghana is of clinical relevance to foster education and promotion of PA. In Ghana, pregnant women believe the “myth” (mostly in rural areas and underdeveloped regions) that exercising in the first trimester might lead to miscarriage. Thus, the main objective of this study was to investigate the extent of knowledge and participation levels in PA among pregnant women in Ho, Ghana using a self-developed questionnaire which consisted of some questions adapted from the PPAQ.

**Methods:**

Seventy-seven (*n* = 77) pregnant women between the ages of 18–50 years were recruited from three hospitals across the Ho municipality of Ghana. A self-developed questionnaire which consisted of some questions taken from the PPAQ was administered to participants under the researchers' supervision. Spearman's correlation analysis was used to find the association between the level of participation in PA, knowledge of PA and gestational age among pregnant women.

**Results:**

From the total participants (*n* = 77) recruited, 57 (74%) of the participants scored high in PA knowledge. Most of the participants 48 (62.3%) answered that PA promotes healthy pregnancy. Participants who reported barriers to PA during pregnancy were no exercise habits 51 (66.2%), having no time 17 (22.1%) and fear of miscarriage 9 (11.7%). There was a significant (*p* < 0.05) association between the level of participation and gestational age. No significant (*p* > 0.05) association between the level of participation and knowledge of PA was observed.

**Conclusion:**

There is a high level of knowledge of PA among pregnant women in Ho, Ghana. However, most pregnant women rather engage in PA as their gestational age increases. Thus, to foster sustainable exercise participation during pregnancy, all healthcare providers saddled with the responsibility of providing maternal healthcare must strengthen the education and promotion of exercise and PA among pregnant women in Ho, Ghana.

## Introduction

Physical Activity (PA) is any voluntary movement produced by skeletal muscle contractions which require energy (1). Exercise is PA that is planned, structured and repetitive for conditioning the body (2). Regular participation in PA improves the overall general health and fitness of pregnant women (3). The maternity period is a vital part of the reproductive life of women with many physical and biological changes in the body during pre-post pregnancy (4). PA amongst pregnant women improve posture and decreases discomforts such as backaches and fatigue (5). During pregnancy, physical and biological changes cause hormonal imbalances which lead to anxiety and stress among pregnant women (6). However, PA during pregnancy rather improves mood (7, 8), self-esteem (9, 10), anxiety (11, 12), and depressive disorders (13). Liu et al. (14) suggested exercises usually prescribed for pregnant women include water aerobics, running, brisk walking, yoga, Kegel and breathing exercises. Exercises such as brisk walking, stationary cycling and swimming improve both cardiovascular and muscular systems and have been recommended for pregnant women (14–16). Additionally, special antenatal yoga and pilates designed for expectant mothers reduce stress, improve flexibility, and encourage stretching and focused breathing (17). These exercises are safe and may not expose these expectant mothers to any risks or complications. Furthermore, there is evidence that PA may prevent gestational diabetes, relieve stress, and build more stamina needed for labor and delivery (18, 19). There are certain contraindications to exercise during pregnancy which may include but are not limited to: cervical insufficiency, pre-eclampsia, heart and lung diseases, placenta previa and severe anemia (20, 21). Thus, a clinical decision should be sought to rule out any contraindications to exercise during pregnancy (22, 23).

PA in the general population is considered too low and this is reflected in pregnant women as well (24, 25). The American College of Obstetricians and Gynecologists (ACOG) has suggested that healthy pregnant women should engage in 150 min of moderate PA per week (26, 27). Despite this, there have been some misconceptions associated with PA and exercises during pregnancy including miscarriage, low birth weight, and early delivery (28, 29). In Ghana, pregnant women (mostly in rural areas and underdeveloped regions), believe the myth that exercising in the first trimester might lead to miscarriage (30). Interestingly, barriers to the participation of exercise among pregnant women have been reported and these barriers are a combination of physical, psychological, and social factors (31, 32). Whilst some pregnant women reported embarrassment about their body appearance, others also reported that the lack of social support from loved ones, and the fear of danger of exercise to themselves and the growing fetus are some of the reasons for which they would rather not participate in exercise or PA (32–35). Some studies (32, 34) reported a lack of education and knowledge of safe ways to exercise during pregnancy. It is said that women who exercise regularly before pregnancy are more likely to keep this attitude during pregnancy than those who were sedentary before becoming pregnant (36, 37). Thus, education and health promotion programs targeting women in their reproductive years are of vital importance for achieving sustainable health practices.

The Royal Australian and New Zealand College of Obstetricians and Gynecologists conducted a study to evaluate the knowledge of PA among pregnant women. They reported that most of the participants correctly reported knowledge of safe levels of PA in pregnancy in line with the recommendation for exercise during pregnancy (38). Also, a study conducted in South Africa reported that (70.2%) pregnant women received PA information from television, the radio, and other media sources (39). However, a study conducted in Nigeria on the knowledge and attitude of pregnant Nigerian women toward antenatal exercise reported that (15.8%) of the respondents had a negative attitude toward antenatal exercise due to limited information on exercise (83.3%) and tiredness (70.0%) as another factor (29). In Ghana, Moses et al. (30) reported that (71.5%) pregnant women were aware of the importance of PA in the Kumasi metropolis but concluded that pregnant women do not spend more time performing PA. Existing literature on the association between knowledge of PA and participation in PA among pregnant women are limited especially in sub-Sahara Africa (29, 39). Only one study (30) has been conducted and published in Ghana. The Volta region of Ghana is one of the less developed regions with poor infrastructures and amenities. The Pregnancy Physical Activity Questionnaire (PPAQ) has been validated for use in assessing PA in pregnant women (40, 41). However, implementing the standardized PPAQ can be problematic in context where cultural and health beliefs toward exercising during pregnancy exist. Thus, understanding the knowledge and participation levels in PA of pregnant women in underdeveloped regions in Ghana is of clinical relevance to foster education and promotion of PA. The main objective of this study was to investigate the extent of knowledge and participation levels of PA among pregnant women in Ho, Ghana using a self-developed questionnaire which consisted of some questions taken from the PPAQ.

## Materials and methods

### Study population

Participants were recruited through advertisement *via* posters pasted in the consulting rooms in hospitals and direct word-of-mouth communication of the research topic to the participants. A total of 77 participants with ages between 18 and 50 years were recruited for the study. The study population consisted of pregnant women in Ho, who regularly visited the antenatal clinics of Ho Teaching Hospital, Ho Municipal Hospital and Ho Royal Hospital of Ghana. Exclusion criteria were: pregnant women who had critical health conditions like uncontrolled gestational diabetes, pre-eclampsia, and placenta previa because researchers were aware that such women were advised by healthcare providers to rest and avoid any form of physical stress or strain to prevent complications. Again, pregnant women who were not frequent with their antenatal visits were excluded because researchers had limited time for data collection hence could only use participants who were readily available. Lastly, those below 18 years were also excluded because researchers needed to seek consent from their guardians before administering the questionnaires. Ethical approval for the study was sought from the Research and Ethics Committee of the University of Health and Allied Health Sciences [Protocol Identification Number: UHAS-REC A.8 (11) 20-21] and written informed consent was obtained from all participants.

### Measures

A new self-developed questionnaire which consisted of some questions adapted from the PPAQ were administered to the participants. The questions taken from the standardized PPAQ was necessary for the setting of the current study. The PPAQ has been successfully used in studies published elsewhere (40, 41). The new self-developed questionnaire was administered to participants to fill to assess their knowledge and participation levels in PA. Data collection started from March 16^th^ to May 12^th^ of 2021. Caution was taken to prevent COVID-19 infection. Chasan-Taber et al. (41) developed the PPAQ with 32 activities including household/caregiving (13 activities), occupational (five activities), sports/exercise (eight activities), transportation (three activities), and inactivity (three activities), respectively. The new self-developed questionnaire consisted of 25 questions which were divided into three (3) sections. Section one consisted of 8 questions on socio-demographic information [Adapted PPAQ (*n* = 0, 0%) vs. New self-developed questionnaire (*n* = 8, 100%)]. The second section consisted of 5 questions on knowledge of PA, positive role of PA during pregnancy, and the source of information on PA [Adapted PPAQ (*n* = 0, 0%) vs. New self-developed questionnaire (*n* = 5, 100%)]. Section three of the questionnaire consisted of 12 questions focusing on household/caregiving activities [Adapted PPAQ questions (*n* = 6, 100%) vs. New self-developed questionnaire (*n* = 0, 0%)], occupational activities [Adapted PPAQ (*n* = 2, 66.7 %) vs. New self-developed questionnaire (*n* = 1, 33.3%)], sports/exercise activities [Adapted PPAQ (*n* = 0, 0%) vs. New self-developed questionnaire (*n* = 1, 100%)], barriers to PA [Adapted PPAQ (*n* = 0, 0%) vs. New self-developed questionnaire (*n* = 1, 100%)], and benefit of PA during pregnancy [Adapted PPAQ (*n* = 0, 0%) vs. New self-developed questionnaire (*n* = 1, 100%)] (see Supplementary Table S1).

### Sample size

The sample size was determined using records of attendance of the pregnant women visiting the antenatal clinics from the various hospitals. The total amount of pregnant women attendance to the antenatal clinic from August 2020–February 2021 was 96. The Slovin's formular was used for calculating sample size for the study with a confidence interval of 95% and *p* = 0.05 (42). The sample size of (*n* = 77) was obtained using the Slovin's formular below.


$$\begin{array}{l} n=\frac{N}{1+Ne^{2}} \end{array}$$

where “*n*” is the sample size, “*N*” is the population size and “*e*” is the margin error.


$$\begin{array}{lll} N & = & 96\;and\;e=0.05\\ n & = & \frac{96}{1+{96\left(0.05\right)}^{2}}\\ n & = & \;77.4 \end{array}$$

### Statistical analysis

Data were analyzed using Jamovi version 1.6.23 software. Descriptive statistics was used to report the demographics and estimate the level of knowledge and participation in PA. Data were represented in frequency tables. Spearman's correlation analysis was used to find the association between the level of participation in PA, knowledge of PA and gestational age among pregnant women. The level of significance was set at *p* < 0.05.

## Results

### Participant demographics

There were (*n* = 77) participants in this study. The demographic data are presented according to the following variables: age, relationship status, educational level, occupation, planned pregnancy, antenatal care type and previous history of abortion (see Table 1). Most participants were between the ages of 21–30, (*n* = 41, 53.3%) and 31–40, (*n* = 34, 44.2%) whereas (*n* = 2, 2.6%) were age of <20. Of the total participants; (*n* = 10, 13.0%) were single whilst (*n* = 67, 87.0%) were married. The highest level of education of participants were: primary (*n* = 13, 16.9%), JHS (*n* = 5, 6.5%), SHS (*n* = 22, 28.6%) and Tertiary (*n* = 37, 48%). Occupations of the participants were: government staff (*n* = 27, 35.1%), private sector workers (*n* = 36, 46.8%), and traders (*n* = 5, 6.5%) whilst (*n* = 4, 6.5%) were students and (*n* = 5, 5.3%) were unemployed. 56 (72.7%) of the participants planned their pregnancies, whilst 21 (27.3%) were unplanned. The antenatal care types of the participants were public hospital (*n* = 54, 70.1%) and private hospital (*n* = 23, 29.9%), respectively. Regarding the history of abortion, (*n* = 7, 9.1%) of participants had a history of abortions whilst (*n* = 70, 90.9%) had none (see Table 1).

**Table 1:** Profile of participants by age, educational level, relationship status, occupation, planned pregnancy, antenatal care type and history of abortion (*n* = 77).

**Variable**	**Frequency (*N*)**	**Percentages (%)**
**Age ranges**		
<20	2	2.6
21–30	41	53.2
31–40	34	44.2
**Educational level**		
Primary	13	16.9
JHS	5	6.5
SHS	22	28.6
Tertiary	37	48.0
**Relationship status**		
Single	10	13.0
Married	67	87.0
**Occupation**		
Government staff	27	35.1
Private worker	36	46.7
Trader	5	6.5
Student	4	5.2
Unemployed	5	6.5
**Planned pregnancy**		
Yes	56	72.7
No	21	27.3
**Antenatal care type**		
Private	23	29.9
Public	54	70.1
**History of abortion**		
Yes	7	9.1
No	70	90.9
**Gestational age**		
First trimester	50	64.9
Second trimester	17	24.7
Third trimester	8	10.4

JHS, Junior High School; SHS, Senior High School.

### Knowledge of physical activity

A set of questions that required a “Yes” or “No” answering system was used to assess the level of PA knowledge among the participants, where “1” or “0” were scored, respectively. Four questions were asked in this section to ascertain if participants knew PA and exercise. First, (90.9%) responded “Yes” whilst (9.1%) responded “No” on ‘if they had ever heard of PA during pregnancy. Secondly, 66 (85.7 %) responded “Yes” whilst 11 (14.3 %) responded “No” “if they know that PA has a positive role in pregnancy”. Also, most of the participants 64 (83.1 %) said “Yes” to “Do you think PA can reduce pregnancy-related complications” whilst the remaining 13 (16.9 %) said “No.” Lastly, participants were asked “if they recommend PA during pregnancy” and 69 (89.6 %) said “Yes” whilst 8 (10.4 %) said “No” (see Table 2).

**Table 2:** Participants' responses to questions on knowledge of physical activity (*n* = 77).

**Item**	**Yes (%)**	**No (%)**
Have you ever heard of physical activity during pregnancy?	70 (90.9 %)	7 (9.1 %)
Does physical activity have a positive role in your pregnancy?	66 (85.7 %)	11 (14.3 %)
Do you think physical activity can reduce pregnancy-related complications?	64 (83.1 %)	13 (16.9 %)
Do you recommend physical activity during pregnancy?	69 (89.6 %)	8 (10.4 %)

### Source of knowledge

As follow-up questions, participants were given three options of possible sources of information about PA and pregnancy and the following were their responses: Health facility 48 (68.4%), media 17 (24.3%), and friends 5 (7.1%) (see Table 3).

**Table 3:** Sources of knowledge of participants on physical activity.

**Variable**	**Frequency**	**Percentages**
	**(*N* = 70)**	**(%)**
**From where did you hear about physical activity during pregnancy?**		
Health facility	48	68.4
Media	17	24.3
Friends	5	7.1

### Level of knowledge

To be able to assess the overall level of knowledge of PA among participants, a set of questions that also required a “Yes” or “No” answering system was used. These questions covered basic household, occupational and leisure time activities. A set of 10 questions with individual score values of 1 for a “Yes” response and 0 for a “No” response was asked. The cumulative scores of the responses were graded as High for a score of (8–10), Moderate for a score of (5–7), and Low for a score of (0–4). Each participant was required to score between 0 and 10 which upon grading represented their level of PA. 57 (74.0%) of the participants scored “High,” 14(18.2%) scored “Moderate,” and 6 (7.8%) scored “Low” for the levels of knowledge of the PA category (see Table 4). No significant (*r* = 0.061, *p* > 0.05) association between the level of participation and knowledge of PA was observed. This shows that level of knowledge does not increase the level of participation in PA (see Table 5). There was a significant (*r* = 0.280, *p* < 0.05) association between level of participation and gestational age. This shows that pregnant women engage in PA as their gestational age increases (see Table 6).

**Table 4:** Participants' levels of knowledge of physical activity (*n* = 77).

**Level of knowledge**	**Frequency (*N*)**	**Percentage (%)**
High	57	74.0
Moderate	14	18.2
Low	6	7.8

**Table 5:** Correlation analysis between knowledge of PA and level of participation of PA in pregnant women.

**Variable**	**Value**	**Knowledge of PA**	**Level of participation of PA**
Knowledge of PA	r	1	0.061
	*P*-value		0.600
Level of participation of PA	r *P*-value	0.061 0.600	1

PA, physical activity. *p*-value was set at 0.05.

**Table 6:** Correlation analysis between the level of participation of PA and gestation age of pregnant women.

**Variable**	**Value**	**Gestational age**	**Level of participation of PA**
Gestational age	r	1	0.280
	*P*-value		0.014*
Level of participation of PA	r	0.280	1
	*P*-value	0.014*	

PA, physical activity. ^*^Significant at p < 0.05.

### Type of physical activity

Some basic PA were listed in Table 7 for participants to select the type of activities they engage in to assess their level of participation in PA and antenatal exercises. The same scoring method to assess the level of knowledge of PA was used here to classify whether the level of participation was high, moderate, or low. 59 (76.6%) scored “High,” 15 (19.5%) scored “Moderate,” and 3 (3.9%) scored “Low” (see Table 8).

**Table 7:** Participants' responses to questions on the type of PA (*n* = 77).

**Item**	**Yes (%)**	**N0 (%)**
1. Do you partake in general cleaning?	72 (93.5)	5 (6.5)
2. Do you care for a child or an elderly person?	49 (63.6)	28 (36.4)
3. Do you go shopping in the market?	68 (88.3)	9 (11.7)
4. Do you partake in general kitchen activities?	75 (97.4)	2 (2.6)
5. Do you wash clothes by hand?	71 (92.2)	6 (7.8)
6. Do you organize your room or make your bed?	75 (97.4)	2 (2.6)
7. Do you move and lift light loads?	57 (74.0)	20 (26.0)
8. Do you scrub your bathroom?	57 (74.0)	20 (26.0)
9. Do you go to work and perform your job tasks?	62 (80.5)	15 (19.5)
10. Do you perform antenatal exercises such activities as walking and stretching during your leisure time?	68 (88.3)	9 (11.7)

**Table 8:** Participants' levels of participation in physical activity (*n* = 77).

**Level of participation**	**Frequency (*N*)**	**Percentages (%)**
High	59	76.6
Moderate	15	19.5
Low	3	3.9

### Barriers to physical activity

As part of assessing the participants' level of participation in PA, participants were asked about the barriers to participating in antenatal exercises. Participants reported barriers to PA during pregnancy were no exercise habits 51 (66.2%), no time 17 (22.1%) and fear of miscarriage 9 (11.7%) (see Table 9).

**Table 9:** Responses on barriers to participating in antenatal exercises (*n* = 77).

**Variable**	**Frequency**	**Percentage**
	**(*N*)**	**(%)**
**Which of the following is a barrier to not participating in antenatal exercises?**		
Having no exercise habits	51	66.2
Having no time	17	22.1
Fear of miscarriages	9	11.7

### Benefits of exercise during pregnancy

The participants were asked why PA during pregnancy is beneficial. 48 (62.3%) of the participants reported “it promotes healthy pregnancy,” 23 (29.9%) reported “it facilitates delivery” whilst the remaining 6 (7.8%) of the participants reported “it reduces pregnancy-related complications” (see Table 10).

**Table 10:** Participant's specific responses to why physical exercise during pregnancy is beneficial (*n* = 77).

**Variable**	**Frequency**	**Percentage**
	**(*N*)**	**(%)**
Promotes healthy pregnancy	48	62.3
Facilitates delivery	23	29.9
Reduces pregnancy-related complications	6	7.8

## Discussions

The main aim of the current study was to evaluate the knowledge and participation levels of PA among pregnant women in Ho, Ghana using a self-developed questionnaire which consisted of some questions adapted from the PPAQ. There was a 100% response rate for this study, the study population constituted participants from a wide range of pregnant women (from both public and private hospitals) from different age groups (i.e., 18–50 years) giving a good representation of the population. Per the findings of this study, majority (74.0%) of the study participants had high knowledge of PA. This is similar to the results reported by Moses et al. (30) in the Ashanti region of Ghana. However, Sujindra et al. (43) and Mbada et al. (29) both reported an overall low level of knowledge of PA among study participants. These contrasting results between this study and the previous studies were due to the different PA and exercise questionnaires consisting of specific knowledge about antenatal exercise variables such as breathing, back care, abdominal exercises, and aerobics exercises such as swimming and cycling during pregnancy were assessed. However, the questionnaires used in this current study consisted of basic household chores and focused on whether the participants had ever heard of PA during pregnancy, whether it had a positive role in their pregnancy, whether they thought it could reduce pregnancy-related complications and whether they would recommend PA during pregnancy to others.

It should be indicated that in the study by Mbada et al. (29) among Nigerian women, nearly 70% were employed and had undergone tertiary education, whiles 48.1% of the current study subjects had completed tertiary education. Additionally, the lack of education and knowledge about safe exercise practices during pregnancy can be a barrier to exercise (31, 32, 34) and this could prevent pregnant women to begin and sustain exercise practicing during and after pregnancy. The results indicated that (76.6%) of the study participants had a high level of participation in PA, whiles (19.5%) had a moderate level of participation in PA. Participants were asked objectively whether they thought PA during pregnancy was beneficial and the responses were as follows; 62.3% of the participants said that it promotes healthy pregnancy whilst 29.9% said that it facilitates delivery. Okafor and Ter Goon (39) reported in their study that; the majority of their participants' reported PA was beneficial in improving cardiovascular function (86.3%) and reducing infant weight (61.4%), reducing musculoskeletal discomfort (82.7%), reducing back pain (85.7%) and incidence of muscle cramps and oedema in the lower limbs (91.3%). These findings were synonymous to data from the current study and show that PA is beneficial during pregnancy. From the results, (68.6%) of the study participants heard about PA from health facilities whilst (24.3%) reported hearing about PA during pregnancy *via* the media. In contrast, Okafor and Ter Goon (39) reported that (70.2%) of their study participants heard of PA from the media whilst (27.0%) heard it from their friends. This shows that social media and social support can be used as an avenue for exercise participation and health promotion (44). Additionally, using telemedicine as a form of exercise guidance has been reported to significantly lower premature delivery (45).

According to the findings of this study, the barriers for not engaging in PA by participants were no exercise habits, having no time and fear of miscarriage which was similar to the findings of Okafor and Ter Goon (39). Muzigaba et al. (46) recorded similar results as participants reported fear of hurt for baby and mother as well as no time as barriers to engaging in PA during pregnancy. Unsupportive environment as well as not knowing when and how to participate in PA were also recorded as barriers (46). This shows that education and sustainable exercise programs structured by healthcare providers especially exercise professionals are needed to facilitate PA participation among pregnant women. Likewise, education to the general public on supporting and encouraging expectant mothers should be considered, and to promote social support to these women. PA and exercise training should also be included in the education given at antenatal clinics to increase the knowledge of PA during pregnancy. This will help pregnant women to feel confident and engage in exercise participation which has been proven to be beneficial for pregnancy outcomes (8, 29, 45, 47).

Furthermore, the results indicated no significant (*p* > 0.05) association between the level of knowledge of PA of the participants and their level of participation in PA. On the contrary, Abedzadeh et al. (48) reported a significant association between their participants' knowledge and PA participation during pregnancy. Also, the significant association (*p* < 0.05) between the gestational age of the participants and their levels of participation in PA suggested that pregnant women would rather engage in PA as their gestational age increases in the current study. Conversely, it was reported by Watson et al. (49) that pregnant urban black South African women decreased their participation in PA from the second to third trimester. This is interesting and noteworthy because one would think that women living in the urban areas are more informed on the health benefits of PA during pregnancy due to the improved health infrastructures and access to information as compared to those in the rural areas (50). Again, uncertainty still remains in linking educational background to the level of PA participation. This could be a contributing factor as Watson et al. (49) reported that majority of the participant's highest level of education was secondary level, whereas that of this study was at the tertiary level. Ultimately, women should be encouraged to cultivate an active lifestyle even before pregnancy to ensure that this behavior is practiced, during the pregnancy period, and thereafter to guarantee a reduction in pregnancy-related complications. Abedzadeh et al. (48) stated in their study that, (48.7%) of pregnant women had continued to exercise till the 9th month of their pregnancy. Interestingly, active women continue to exercise even during pregnancy more than non-active women before becoming pregnant (36, 37).

The standardized PPAQ has been validated to use in assessing PA in pregnant women (40, 41). Whilst the standardized PPAQ may create uniformity among studies assessing PA in pregnant women, it may be difficult to apply the standardized PPAQ in a cultural context where there exist cultural and health beliefs toward exercise during pregnancy. But this current new self-developed questionnaire consisting of some questions adapted from the PPAQ can be the first step and a simple tool to be used in assessing PA in pregnant women in less developed regions of Ghana, and especially in rural areas that believe in a myth that exercising during the first trimester might lead to miscarriage (30). This simple questionnaire could be used in epidemiological studies to assess the knowledge and level of PA in pregnant women in the other less developed regions of Ghana to help develop PA programs for pregnant women as well as women in their reproductive ages. In the clinical settings, healthcare professionals can use this simple questionnaire to assess the knowledge and level of participation of PA in pregnant women, especially in rural and less developed regions of Ghana. Thus, to foster sustainable exercise participation during pregnancy, decision-making bodies, stakeholders, and healthcare providers saddled with the responsibility of providing maternal healthcare must strengthen the education and promotion of PA among pregnant women in Ho, Ghana.

### Strengths and limitations

This is the first study to assess the knowledge and level of PA of pregnant women in Ho, Ghana. The study adds to the body of literature on how the knowledge and level of PA can be assessed in pregnant women in a less developed region of Ghana. In the clinical settings, healthcare professionals can use this simple questionnaire to assess the knowledge and level of participation of PA in pregnant women especially in rural and underdeveloped regions of Ghana. Thus, setting the foundation for education and promotion of PA programs for pregnant women in less developed regions in Ghana. Despite these strengths, the current study had several limitations. Pregnancy is considered sacred and so only a few participants were willing to participate in the current study. Future studies implementing longitudinal research design with a large sample size are warranted. There was limited availability of published materials, especially within Ghana and the Sub-Saharan African continent, for possible comparison of results. Lastly, care should be taken when interpreting the results, as questionnaires may lead to over-estimation of reporting by participants (51, 52). The PPAQ is validated for assessing PA in pregnant women. The current new self-developed questionnaire consisting of some questions adapted from the PPAQ needs validation.

## Conclusion

There is a high knowledge of PA amongst pregnant women in Ho, Ghana. However, most pregnant women rather engage in PA as their gestational age increases. Thus, to foster sustainable exercise participation during pregnancy, decision-making bodies, stakeholders, and healthcare providers saddled with the responsibility of providing maternal healthcare must strengthen the education and promotion of PA among pregnant women in Ho, Ghana.

## Data availability statement

The original contributions presented in the study are included in the article/Supplementary material, further inquiries can be directed to the corresponding author.

## Ethics statement

The studies involving human participants were reviewed and approved by Research and Ethics Committee of the University of Health and Allied Health Sciences [Protocol Identification Number: UHAS-REC A.8 [11] 20-21]. The patients/participants provided their written informed consent to participate in this study.

## Author contributions

DA designed the study. DA and FO wrote and revised the manuscript. FA-S and VN collected the data and conducted the analyses. All authors provided critical feedback, contributed to the final manuscript, and approved the final version of the manuscript.

## Conflict of interest

The authors declare that the research was conducted in the absence of any commercial or financial relationships that could be construed as a potential conflict of interest.

## Publisher's note

All claims expressed in this article are solely those of the authors and do not necessarily represent those of their affiliated organizations, or those of the publisher, the editors and the reviewers. Any product that may be evaluated in this article, or claim that may be made by its manufacturer, is not guaranteed or endorsed by the publisher.
