# Supplementary material for: Knowledge and participation in exercise and physical activity among pregnant women in Ho, Ghana
Source: Front Public Health. 2022 Nov 16;10:927191. doi: 10.3389/fpubh.2022.927191 (PMC9709326; doi:10.3389/fpubh.2022.927191)
Supplement: Supplementary file 1 [file Data_Sheet_1.PDF]

Supplementary table 1. Self-developed questionnaire adapted from the PPAQ (Chasan-Taber et al., 2004)

**Participant ID**…………………………………………………………………………
**SECTION 1: SOCIO-DEMOGRAPHICS**
**1. Age**………………………………………………………………………………
**2. Relationship status**
(a) Single
(b) Widowed
(c) Married
(d) Divorced
**3. Educational Level**
(a) Primary
(b) JHS
(c) SHS
(d) Tertiary
**4. Occupation**
(a) Government staff
(b) Private sector
(c) Unemployed
(d) Student
(e) Trader
**5. Is your pregnancy planned**
(a) Yes
(b) No
**6. Antenatal Care Type**

(a) Private
(b) Public
**7. Previous history of abortion**
(a) Yes
(b) No
**8. What stage of pregnancy are you in?**
(a) First Trimester
(b) Second Trimester
(c) Third trimester
**SECTION 2: KNOWLEDGE OF PHYSICAL ACTIVITY**
1. **Have you ever heard of physical activity during pregnancy**
(a) Yes
(b) No
**2. From where did you hear about physical activity during pregnancy?**
(a) Health facility
(b) Media
(c) Friends
**3. Does physical activity have a positive role in pregnancy?**
(a) Yes
(b) No
**4. Do you think physical activity can reduce pregnancy-related complications?**
(a) Yes
(b) No
**5. Do you recommend physical activity during pregnancy?**
(a) Yes
(b) No
**SECTION 3: PARTICIPATION IN PHYSICAL ACTIVITY**

**1. Do you partake in general cleaning?**	
(a) Yes	
(b) No	
**2. Do you care for a child or an elderly person?**	
(a) Yes	
(b) No	
**3. Do you go shopping in the market?**	
(a) Yes	
(b) No	
(a) 4. Do you partake in general kitchen activities	
(b) No	
**5. Do you wash clothes by hand?**	
(a) Yes	
(b) No	
**6. Do you organize your room or make your bed?**	
(a) Yes	
(b) No	
**7. Do you move and lift light loads?**	
(a) Yes	
(b) No	
**8. Do you scrub your bathroom**	
(a) Yes	
(b) No	
**9. Do you go to work and perform your job tasks?**	
(a) Yes	
(b) No	
**10. Do you perform antenatal exercises such as walking and stretching during your leisure time?**	

(a) Yes
(b) No
**11. If your answer was No, which of the following is your barrier for not participating in antenatal exercise?**
(a) Having no exercise habits
(b) Having no time
(c) Fear of miscarriage
**12. Why is physical activity during pregnancy necessary?**
(a) Promotes healthy pregnancy
(b) Facilitates delivery
(c) Reduces pregnancy-related complications
